# Maze learning by a hybrid brain-computer system

**DOI:** 10.1038/srep31746

**Published:** 2016-09-13

**Authors:** Zhaohui Wu, Nenggan Zheng, Shaowu Zhang, Xiaoxiang Zheng, Liqiang Gao, Lijuan Su

**Affiliations:** 1 College of Computer Science and Technology, Zhejiang University, China; 2 Qiushi Academy for Advanced Studies, Zhejiang University, China; 3 Research School of Biology, the Australian National University, Australia; 4 Department of Biomedical Engineering, Zhejiang University, China

## Abstract

The combination of biological and artificial intelligence is particularly driven by two major strands of research: one involves the control of mechanical, usually prosthetic, devices by conscious biological subjects, whereas the other involves the control of animal behaviour by stimulating nervous systems electrically or optically. However, to our knowledge, no study has demonstrated that spatial learning in a computer-based system can affect the learning and decision making behaviour of the biological component, namely a rat, when these two types of intelligence are wired together to form a new intelligent entity. Here, we show how rule operations conducted by computing components contribute to a novel hybrid brain-computer system, i.e., ratbots, exhibit superior learning abilities in a maze learning task, even when their vision and whisker sensation were blocked. We anticipate that our study will encourage other researchers to investigate combinations of various rule operations and other artificial intelligence algorithms with the learning and memory processes of organic brains to develop more powerful cyborg intelligence systems. Our results potentially have profound implications for a variety of applications in intelligent systems and neural rehabilitation.

Learning is an essential cognitive skill for biological brains from primates to invertebrates^1^,^2^,^3^,^4^. For example, the honeybees, even with a relatively simple nervous system, can learn to navigate in complex mazes^5^. Modern computers with advanced artificial intelligence can also apply learning algorithms to solve problems in the environment^6^. Facilitated by current research in neural signal recording and processing^7^,^8^ and micro stimulation^9^, brains and machines are being interconnected with each other more tightly than ever, resulting in sensory^10^, memory^11^, and motor function rehabilitation or enhancement^11^,^12^,^13^, animal robots^9^,^14^,^15^,^16^,^17^,^18^ and cognitive robotics embodied with biological brains^19^,^20^,^21^,^22^. Such a trend of biological and artificial intelligence integration engenders the question whether hybrid systems possess superior learning ability over their purely biological component. However, to our knowledge, no study has yet demonstrated enhanced learning of hybrid brain machine systems^23^ in maze navigation tasks. Here, we ask whether the way biological organisms learn and make decisions could be altered by enhancing their brains with *machine rule learning*, and how applying the acquired rules by machines affects the learning ability of these hybrid systems.

To answer these questions, ratbots were designed and constructed as a hybrid brain machine system comprised of four components depicted in Fig. 1a,b: an adult Sprague-Dawley rat with electrodes implanted at the bilateral medial forebrain bundles (MFB), a backpack stimulator providing electric stimuli to the rat brain, a camera capturing the movement or the surrounding environment of the rat, and a computer generating stimulation parameters to the backpack stimulator wirelessly. Driven by the video inputs from the camera, learning algorithms and rule operations running on the computer (refer to section Method- “Rule learning algorithms”) calculated the MFB stimulation parameters (the voltage, frequency, duty circle and the number of pulses, see Fig. 2) to guide the rat. The ratbot performed learning tasks autonomously without any human intervention.

We designed a series of training tasks to examine the ratbots’ learning ability in a complex maze as shown in Figs 1c and 3. In each *trial*, the subject had to travel through the maze and make choices at all the six decision points to obtain a water reward at the target unit (i.e., the exit unit, see water reward unit at Fig. 1c for the current trial). It took several consecutive trials for a subject to learn the correct path of the maze in a *session*. The criteria for learning the correct path are that a subject can make the correct choice at least five out of six. If the rats/ratbots were able to navigate along the correct path for at least 3 consecutive trials, the subjects were determined to have learned the task in the current session (and for the current maze as well).

To exclude the subjects’ prior memory, a new maze was constructed for each new session. Six ratbots were used as the subjects to investigate three kinds of rule operations conducted by the computer integrated into the hybrid system, including rule learning, rule application, and rule combination and transfer:

- Rule learning. For ratbot V1 in the task T1, the maze configuration is unknown for the subject. The computer will track the navigation path of the rat and store the traversed units. With the reinforcement learning algorithm (i.e., Q-Learning), the computer will solve the maze by dynamic programming after the first trial and build the reward map based on the Q-table. The computer will extract the reward-increment rule from the reward map.
- Rule application. For ratbot V2 in task T2, the subject applies the predefined rule of landmark following. If the head-mounted mini-camera detects the landmark denoting the correct choice of the decision making point, the computer will deliver the MFB stimulus to inform the rat to navigate along the path denoted by the landmark.
- Rule combination and transfer. For ratbot V2 in task T3, the computer combines the reward-increment rule and the land-mark following rule into a new one. That is, once the head-mounted camera detects the landmark, the computer will increase the level of the MFB stimuli and guide the rat along the direction denoted by the landmark.

The session order of the three experiments in the test was randomized to exclude the experience inequality. Before the series of maze learning tasks, the rats were trained in the mazes to guarantee equal navigation experience among subjects for the three maze learning tasks. We collected experiment data of 16, 16, 18, 16, 15 and 18 sessions from the task T1, T1 Control, T2, T2 Control, T2 with mask, T3 respectively (denoted as N in Fig. 4). In each session, the subjects learned the maze with eight trials.

## Results

**Figure 1:**
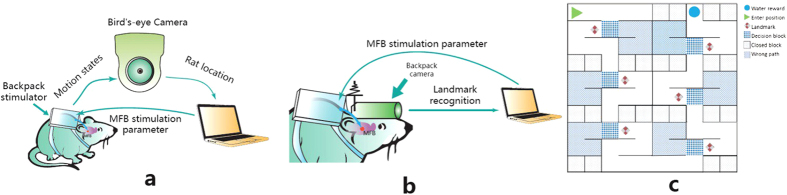
Ratbot and maze. (**a**) The components of V1 ratbots. The rat was implanted with stimulating electrodes at the Medial Forebrain Bundle. The backpack stimulator mounted on the back of the rat received MFB stimulation parameters from the computer, and delivered stimulation pulses into the rats’ brains. A bird’s-eye camera monitored the rats’ movement and position. The detailed structure of the maze and the bird camera is depicted in Section “Method” and shown in Fig. 3. (**b**) The components of V2 ratbots. The hardware of V2 ratbots was the same as that of V1 ratbots, except that the former carried a head-mounted mini-camera instead of a bird’s-eye camera. The mini-camera transmitted video frames wirelessly to the computer which then tried to recognize the landmarks. (**c**) Maze. The maze was composed of 10 × 10 units (each of 15 cm × 15 cm × 15 cm). All of the surrounding walls of the units were removable, making the maze reconfigurable with an arbitrary formation. In our experiment, the maze was designed to consist of six decision points. The correct directions of the six decision points could be randomly assigned as left or right, resulting in up to 64 different maze configurations.

We first examined *whether a ratbot could construct a digital spatial map and extract a MFB reward rule from the reward map of the maze*. The subjects were the V1 ratbots shown in Fig. 1a and a bird’s-eye camera tracked the rats’ instantaneous position and movement. During the rats’ navigation in the maze, the Q-Learning algorithm running on the computer calculated graded reward levels to the visited positions and thus generated a digital reward map iteratively in the learning trials (the algorithm details is presented in subsection “Rule learning algorithms” and “Graded level stimulation” in “Methods”). According to the rat’s position and the reward map, the computer produced real time MFB stimulation parameters sent to the backpack stimulator and thus affected the rat’s exploration in the maze. When the ratbots got closer to the target unit (i.e., the water reward), the rat received the higher intensity of MFB stimulation from the computer. This experiment was denoted as the task T1.

The corresponding control experiments (T1 Control) were conducted using the same rats as in the task T1 with the implanted electrodes, but without any computing device. The rats explored the maze only depending on their biological sensation and spatial memory, without the computer’s involvement. As shown in Fig. 4a, the percentages of correct choices for the task T1 Control started from approximately 50%, indicating random choices when the unenhanced rats had no a *priori* spatial memory of the maze. The control subjects learned *the correct path* (at least five correct choices out of all six in the maze) on the 6th trial on average, much slower than the 3rd trial of V1 ratbots in the task T1.

With graded-level MFB stimuli, V1 ratbots displayed an accelerated trend of learning over the control subjects, providing evidence of learning enhancement by artificial intelligence. Once a V1 ratbot completed a session in a given maze, a digital reward map for this maze was created (details see subsection “Rule learning algorithms” in “Methods”). The reward maps of three ratbots in their first sessions were analyzed to extract an incremental-reward rule by the RULES algorithm^24^: there is a monotonic increment of MFB stimulation level along the correct path to the target unit from the starting unit. Moreover, the reward maps of the rest sessions were used to validate the extracted rule.

### Can ratbots learn to apply a rule to navigate the maze?

Experiment task T2 was designed to investigate whether ratbots can apply the rule of following a set of landmarks and obtain enhanced learning performance over the control rats. Six landmarks were placed into the maze, each representing the correct direction at the corresponding decision point as shown in Fig. 1c for a specific session (as mentioned above, the maze is configured for each session to exclude the prior spatial memory). To recognize the landmarks from the visual field of the rat, the bird’s-eye camera was replaced by a mini-camera mounted on the rat’s head, i.e., V2 ratbot in Fig. 1b. Once the landmark was detected by the camera, a constant level of electric MFB stimuli was delivered to the ratbot to indicate the correct direction. The control experiments with landmarks in the mazes (T2 Control) were conducted with the unenhanced rats to provide the baseline learning performance.

As shown in Fig. 4b, V2 ratbots in the task T2 outperformed the unenhanced rats in the task T2 Control by a large extent, with the number of trials to complete learning the maze decreasing from 5 to 2. Even when the rats’ biological vision and the tactile sensation from the whiskers were blocked by a face mask (experiments denoted as the task T2 with mask), the subjects still displayed a similar navigational ability to those of V2 ratbots without face masks in the task T2 as shown in Fig. 4b. There was no significant difference between the results in the task T2 (normal biological sensation) and the task T2 with mask, indicating that machine intelligence can make up for the loss of normal sensory input. The p-value of the average performance across trials 1–4 of T2-T2 with mask is 0.968, and trials 5–8 is 0.469.

### Can ratbots conduct the rule combination and transfer rules to new mazes?

With the reward increment rule learned by ratbots in the task T1 and the landmark following rule applied in the task T2 or the task T2 with mask, we also were able to demonstrate the combination and transfer of rules into novel mazes. In the task T3, the reward increment rule and the landmark following rule were combined into a new rule that when the mini-camera caught sight of a landmark (i.e., the ratbot was on the correct path), the reward level of the MFB electrical stimulation was increased after the ratbot passed the landmark. To capture the landmarks in the learning process, the V2 ratbots were used as the subjects in the task T3. With the combined rule, the ratbots reached an average performance of approximately 80% at the first trial and completed learning the maze in just two trials, which demonstrated much faster learning speed compared with the control rats in the task T1 Control and V1 ratbots in the task T1. In the latter two trials (trials 7 and 8 in Fig. 4b), the ratbots’ correct choice rate remained steady at over 90%, whereas the control rats’ performance was more variable, as shown by the standard deviation (represented by the shaded areas on the graphs).

As shown in Fig. 4, ratbots exhibited shallower learning curves (especially in trials 1 to 4) and required less than half the trial numbers of the control rats to reach a high stable performance of over 80%, showing that the ratbots’ learning ability is superior to that of the unenhanced rats. The ANOVA results denoted by the rectangles in the upper part of Fig. 4a,b showed that there was a significant difference between the performances of ratbots and the corresponding control rats in both the first four trials (learning phase) and in the last four trials (plateau phase). The p-value of the average performance across trials 1–4 of T1 vs. T1 Control is 0.001, and trials 5–8 is 0.006 respectively, while the p-value of the average performance across trials 1–4 of T2 vs. T2 Control is 1.98E-7, and trials 5–8 is 0.015.

## Discussion

We focus on the ratbot as a complete system, which combines the computer with the rat that received MFB stimulation. The MFB stimulation acts as a bridge to link the embedded computer system with the rat. Although previous work has shown that MFB electrical stimulation could provide high motivation in learning tasks^25^, our results are the first to provide the proof-of-the-concept research and indicate that, by graded level MFB stimulation, rule operations and reinforcement learning algorithms running on computing components could affect the learning and decision making process of the biological components in real-time. Environment information can be detected by electronic sensors (such as the camera in this work) to enhance the ratbot sensations over and above those the control-group rats. The learning and memory of the integrated computing system enhance the ratbot with powerful electronic learning and memory abilities, which is presented as the three series of behavior experiments. We believe that artificial intelligent algorithms will play a complementary and enhancing role in the learning or memory process of the integrated biological brain in the future hybrid brain-computer systems.

Graded levels of MFB stimuli are mapped from the converged result of Q-learning algorithm in the task T1, which is explicitly required by the computer model. While in the task T2 MFB stimulation of a single level was used, in the task T3, the same level MFB stimulation was replaced by the graded levels. The result in Fig. 4b showed that ratbots with the graded level MFB stimulation in the task T3 performs better than the same level in task T2 or T2 with mask. Although the mechanisms and functional significance of reward relativity are still only partially understood, the maze learning experiments illustrated that the incremental stimulation (rewards) could improve the learning ability. This result indicated two folds of possible reasons: incremental rewards for different choices at the decision position may lead to quicker learning about the correct choice; and incremental rewards along the whole navigational path may lead to continuously high motivation to the maze target unit.

Percentage of correct choices and trials number needed to learn the complex maze were presented in the results. The time used to complete each trial is also an important indicator of an online navigation system. For T1 control and T1, the time used to complete each trial was shown in Fig. 5a. The results showed an interesting phenomenon that for the initial several trials the navigation time taken by T1-control is greater than T1, which may indicate that the control rats need more time to learn the path to the target unit than that for the ratbots with the computer-delivered MFB stimulation. And after having learned the navigation task, both ratbots and rats can finish the goal within a short time. Moreover, in the experiments we also find another interesting phenomenon that some rats prefer a pause when they received the MFB stimulus. As shown in Fig. 5b, for the initial several trials, the ratbots with computer-delivered MFB stimulation need less time. However, in the later trials, the ratbots took longer time to finish the task although with higher percentage of correct choices than the control subjects (i.e., rats).

Even though we found significant performance differences between Ratbot groups and control groups, the performance of control groups also increased gradually, which can be partially attributed to the rats’ spatial memory for the navigation path. For T1 Control task, there was no landmark; and there were some landmarks for T2 Control task. Both tasks involved no rules about MFB stimulation. The phenomenon of better performance for T2 Control than T1 Control showed that landmarks at the decision positions improved the animals’ navigation ability. Besides, we conducted additional experiments immediately after the last trial in some sessions of the task T1, T2 and T3, with the backpack of the ratbots removed. It was found that the ratbots without MFB stimulation could still remember the correct path to the target unit after they had finished eight trials in the maze. Consistent with the previous study result^25^,^26^, the graded-level MFB stimulation delivered by the computer algorithm was able to help the rat form a spatial memory of the correct path in the complex maze. This leaves the interesting question of whether the rats have created a cognitive reward map in their brain^27^. If there exists such a reward map in the rat brain, how the computer input affects the memory formation and memory retention need to be examined in future studies.

From the animal learning perspective, animals may associate their behavior with the MFB stimulations to complete the navigation tasks, which also can be explained by the associative learning mechanisms. The MFB stimulation itself could increase the learning rate of the acquisition, even taking no account of the difference between contingent and the noncontingent MFB stimulation. It would be better to do a yoke control to test whether there exists equivalent learning. The overall comparisons conditions and theoretical verification depend on the extensive experiments as the future work, which will provide more evidence of broader cognitive functionalities enhanced and exhibited by the hybrid system.

Future work should also address several aspects on the ratbot model presented in this paper. For example, how long can the learned rules of incremental reward and landmark-following be retained in the memory of the ratbots? Neural signals from the tactile/visual cortex or memory neural circuits of the ratbots could be recorded and decoded to obtain the biological representation of the environments and events. These information could potentially be merged with the input from artificial sensors, such as the cameras in the experimental setup, to build a more precise and comprehensive representation of the maze ‘world’ in the computer. Meanwhile, given advances in feedback methods, besides the virtual MFB reward, more navigation information from the computing results could be delivered into the neural circuits of the ratbots to make the organic brain and computer more tightly integrated. Besides, future work should include how the learning and memory of the computing system promotes the corresponding cognitive ability of the hybrid system. Much more work is needed to clarify other aspects of the computer that affect the process and find the best algorithm. Further works on reinforcement learning algorithms and reinforcement sequences can be explored.

This work describes an experimental model of the novel cyborg intelligent systems and provides the evidence of the proof-of-the-concept. The ratbots clearly demonstrates the excellent behavior performance in the maze learning task, due to the rule operations conducted by the computer. It remains possible that the fruitful achievements in artificial intelligence such as knowledge based system and learning from big data can contribute to stronger computing intelligent components integrated with the biological living brain. Hence, the experiments presented here show great potential to combine the strengths of biological organisms and computing components into hybrid brain-machine systems. Prospective applications include the use of computational power to enhance learning and other cognitive functionalities for animal-robots hybrids or in the rehabilitation of brain-damaged humans, and even for people with high cognitive loads, such as soldiers and astronauts.

## Methods

Adult male Sprague-Dawley rats (weight 250–300 g) were used in the maze learning experiments. All experiments in this study were carried out in accordance with the guidelines for the Care and Use of Laboratory Animals of Zhejiang University, and approved by the Institutional Animal Care and Use Committee of Zhejiang University.

## Rat preparation

The laboratory rats were obtained from the Experimental Animal Center of Zhejiang Academy of Medical Sciences (Certificate No. SCX20030001). Rats were housed under a controlled temperature (24 ± 2 °C), 12 h light and 12 h dark cycle. Before performing the maze learning tasks, the rats were allowed free access to water and food.

All rats were operated on to implant two pairs of stimulating electrodes in the Medial Forebrain Bundle at both sides. During the surgery, rats were anesthetized with an intraperitoneal injection of Pentobarbital Sodium solution (2%, 40 mg/kg) and then fixed in a stereotaxic apparatus (Stoelting Co, Wood Dale, IL, USA). After incision of the scalp at the midline, six holes (1 mm in diameter) were drilled in the skull for inserting of two pairs of stimulating electrodes (insulated nichrome wires, 65 μm in diameter with a 0.5~1 mm vertical tip separation), and for anchoring the stainless steel screws. Using coordinates obtained from the Rat Brain Atlas, the bipolar stimulating electrodes were placed at the MFB (AP −3.8 mm, ML ±1.6 mm, DV +8.2 mm). The electrodes were fixed to the skull with dental acrylic. After the surgery, at least seven days were allowed for the rats to recover.

After the rats had recovered from the surgery, two preliminary tests were performed to select candidate rats for our experiments. Firstly, the rats were stimulated with voltages from 4 V (i.e., LowV) to 10 V (i.e., HighV). If exhibited excited behavior, such as increased whisker twitching with no abnormal behavior, the surgery was deemed successful. Then the rats carrying the backpack (see “Hardware and apparatus”) were placed into an operant conditioning chamber with a pressing bar. Each time the rat pressed the lever, a reward stimulus at the MFB was instantly delivered to the rat. The rats were allowed at least one hour’s time in the chamber to freely explore the chamber and learn to press the lever. If the rat could learn to press the lever after no more than three times of training, it passed the test. Those rats that successfully passed the two tests were chosen as the candidate rats for the maze learning task.

It is the better to select two simulation reference voltages, LowV and HighV, by assessing the stimulus intensity-lever press response curve for each rat individually^28^. And it is also better to use stimulus current than the stimulus voltage when configuring the electrical stimuli. We have monitored the impedance for a month till finished the experiments. The measurements were in the range 300–500 kΩ, keeping the corresponding stimulus intensity relatively constant.

## Hardware and apparatus

### Backpack stimulator

The backpack stimulator received MFB stimulation parameters wirelessly from a computer (Intel i3 4130, 2G DRAM, Windows 7 operating system) via Bluetooth interfaces. It produced and delivered stimulation pulses to the rats’ brains by its connecting electrodes according to the stimulation parameters received. The micro-processor of the backpack stimulator was a Mixed-Signal ISP FLASH MCU (C8051F020, Silicon Labs, USA).

**Figure 2:**
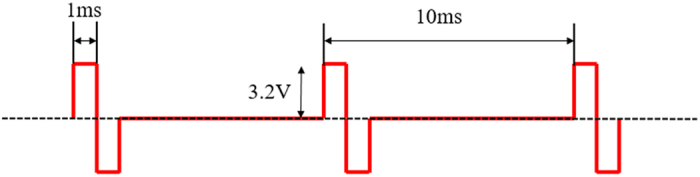
Electric stimulus waves for MFB stimulation. As an example, the frequency of this rectangular biphasic pulses was 100 Hz, generated by a Mixed-Signal ISP FLASH MCU (C8051F020, Silicon Labs, USA) for MFB stimulation. Its voltage, duty circle, and number were set to 3.2 V, 20%, 3, respectively.

For the detailed illustration of the electric stimulus waves, Fig. 2 shows the specification of the characteristics of parameters (for rat No. DH06). A 0.1 s stimulus train of rectangular biphasic (1 ms each phase) pulses at 100 Hz was generated by the backpack and used for stimulation. The initial voltage was set to 3.2 V.

### Camera

**Figure 3:**
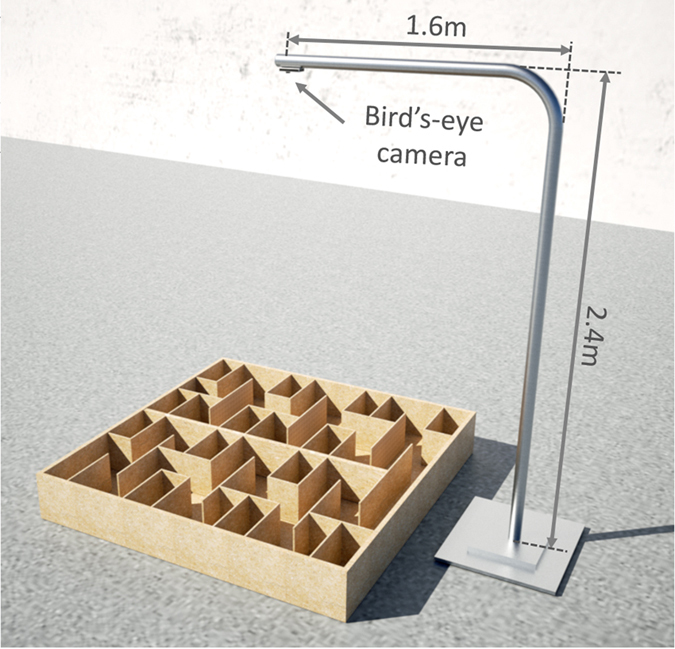
Bird-eye camera over the maze. In order to capture the symmetrical image of the maze, the bird’s-eye camera was placed just above the maze’s center, mounted on the tail of the horizontal arm of an L-shaped steel tripod. The height is 2.4 meter.

**Figure 4:**
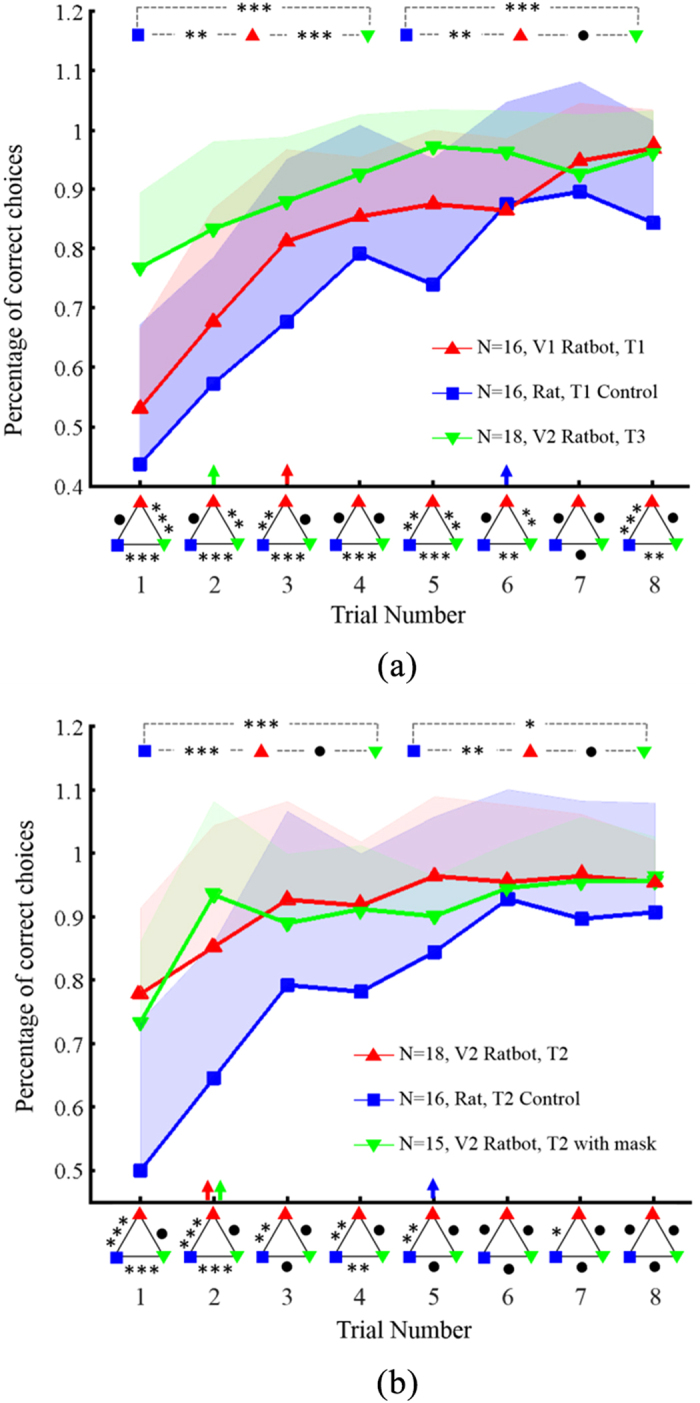
Superior learning performance of Ratbots over unenhanced rats. We collected data in 16, 16, 18, 16, 15 and 18 sessions for T1, T1 Control, T2, T2 Control, T2 with mask, T3 respectively (denoted as N in Fig. 4). The subjects learned one maze in a session with eight trials. One subject was unable to finish all the experiments because of an accidental destruction of the implanted electrodes, resulting in the inconsistent session numbers. (**a**) Learning curves of V1 ratbots in rule learning (T1 in red), control rats in a maze without landmarks (T1 Control in blue), and V2 ratbots with rule combination and rule transfer for new mazes (T3 in green). (**b**) Learning curves of V2 ratbots with rule application of landmark following (T2 in red), control rats in a maze with landmarks (T2 Control in blue), and V2 ratbots with a face mask applying the rule of landmark following (T2 with mask in green). The arrows on the x-axis indicate correct rat choices more than 80% of the time. Standard deviation is depicted by the shaded area. Triangles under the horizontal axis show the results of the ANOVA, that is, whether differences between each pairs of groups were significant in individual trials. The ANOVA results of the average performance across trials 1–4 and 5–8 are also depicted in the upper two rectangles in the figures. All experiments were carried out using the same six subjects as control rat, V1 ratbot and V2 ratbot (the order of experiments conducted can be referred to section Methods “Experiments”). ***denotes significant difference (p < 0.001), **denotes p < 0.05, *denotes p < 0.1 and • denotes no significant difference.

**Figure 5:**
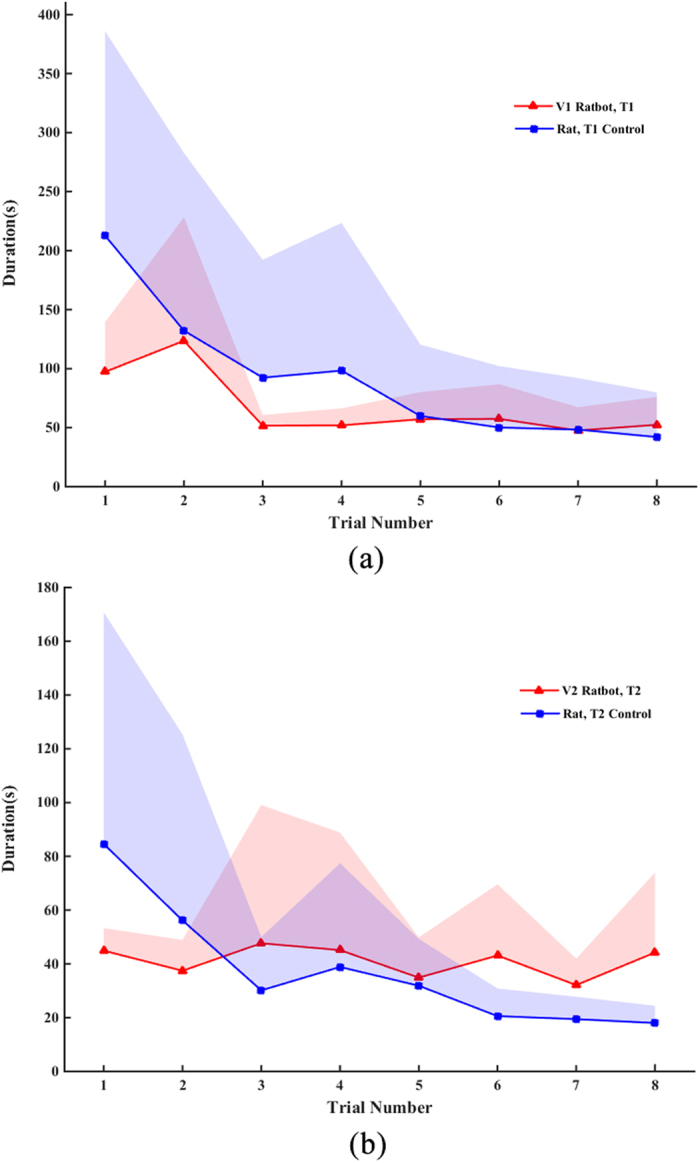
Duration comparison. (**a**) For task T1, in the initial several trials, the navigation time taken by T1-control is greater than T1, which may indicate that the control rats need more time to learn the path to the target unit. (**b**) For task T2, in the initial several trials, the ratbots with computer-delivered MFB stimulation need less time, while in the later trials, the ratbots took longer time.

In our experiments, two cameras were used: one was the bird’s-eye camera and the other was fixed on the head of the subjects. The bird’s-eye camera (C170, Logitech., CH) was wired to the computer and mounted over the maze to monitor the movement and position of the subjects in the maze. As shown in Fig. 3, the bird’s-eye camera was mounted on the tail of the horizontal arm of an L-shaped steel support. In order to capture an overall view of the maze, the camera was placed just above the maze’s center. The distance from our bird’s-eye camera to the maze floor is 2.4 meter.

The mini camera (RS7028C-60, Bangu Tech., CHN) captured real time video frames with the resolution of 640 × 480 pixels and transmitted them wirelessly to the computer. The mini camera was of 7 mm in diameter and 23 mm in length, and was light enough (50 g) for the rat to carry. It was placed on the backpack with its optical axis in the same direction with the head of the rat.

### Maze

A specially designed maze was built for the learning experiments. The maze consisted of 10 × 10 units (each of 15 cm × 15 cm × 15 cm). It was configurable with four removable surrounding walls for each unit. By removing or inserting a surrounding wall, the corresponding two adjacent units became connected or blocked respectively.

As shown in Fig. 1c, the maze can be configured by randomly setting the directions of six decision points, such as LLRRLR, in which L stood for left, and R stood for right. The landmark was a diamond shaped box (6 cm × 6 cm × 1 cm), made of transparent glass, and indicated the correct direction in which to proceed. Papers printed with patterned colors of red and blue were stuck to the inner sides of the landmark to allow visual perception. During experiments, the maze was also covered with a transparent glass plate to prevent the rats from climbing up the maze. Rats were placed into the starting unit for each trial at the beginning. Every decision was a binary choice, in which the rats were obliged to make a choice between two directions at the decision position. The correct directions led the rat to the next decision making point or the reward of water, whereas the wrong one led to a dead end. For different experiments the correct choice for each decision point was randomly distributed.

## Maze learning tasks

### Behavioural training

Before the series of maze learning tasks, the rats that had passed the preliminary tests of MFB stimulation in “Rat preparation” were trained in the mazes to guarantee equal navigation experience among subjects for the preceding maze learning tasks. Before training, the water supply for the rats was deprived for 2 days. During training, the rats were placed at the starting unit, and were allowed to freely explore the maze. Each time when arriving at the water rewarding position, the rats received a small amount of water reward (0.05 mL). Then the rats were brought out of the maze and placed at the starting unit again for the next training. After 15~30 times of such training, the rats should be able to learn the maze task and progress quickly to the water rewarding position (i.e., the target unit) when they were placed into the maze. If the rats were able to navigate along the correct path for at least 3 consecutive trials, they were determined to have learned the task in the current maze. The training procedures were repeated for 5 days and for 20 times each day to train the rats to remember the maze learning task. Once the rat had learned the maze, the maze configuration was changed. The rats that were not active in the maze and could not learn the maze task after the training stage were excluded from the following maze learning experiments.

### Experiments

The V1 ratbot carried a backpack stimulator wirelessly connected to the computer, with a bird’s-eye camera over the maze. For the V2 ratbot, the bird’s-eye camera was replaced with a wireless head-mounted mini-camera. Six rats out of twelve were selected from the aforementioned preliminary MFB tests and behavioural training test. The six rats were used as the experimental subjects for all the tasks conducted in this study. The details of the experimental protocol are listed in Table 1.

The experiments procedure was conducted as follows. The first sessions of T1, T2 and T3 for three ratbots were used to establish the incremental reward rule (T1), landmark following rule (T2), and transfer the rules (T3). Then, the order of the experiments was arranged randomly in the remaining sessions.

### Behavior tracking algorithm

The rat behavior tracking algorithm contains two parts: background subtraction and mass-center extraction. The Background subtraction was used to build a still background model from all or a subset of the frames in a video clip and obtain the foreground objects (i.e. the rat). We used a mixture of Gaussian distributions to model the background independently at each (*x*, *y*) pixel. Using the binary image given by the previous step, the mass center of the rat is extracted using the following equations. The *m*_00_, *m*_10_ and *m*_01_ represent 0^th^ and two 1^st^ moments of the binary image.


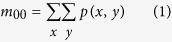



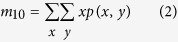



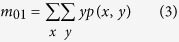


where *p(x*, *y*) represents the pixel set of the rat in the current binary image.

The mass center of the rat, *p*_*mc*_, is computed as follow:


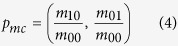


### Rule learning algorithm

**Figure 6:**
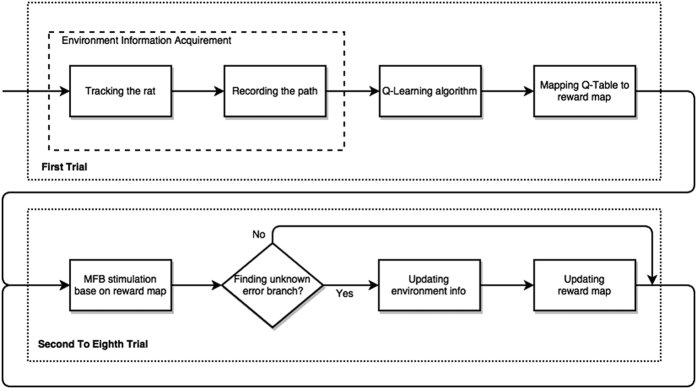
Pipeline of the learning algorithm. Before the first trial of the task T1, there is no prior spatial knowledge about the maze for the ratbots or rats. In the first trial, the computer tracked the rat’s position and recorded the rat’s movement in the maze. When the rat finished the first trial, the environment information of the maze was achieved. The maze problem modeled as a Markov decision process was solved by Q-learning algorithm after the “environment information acquirement” step. After got the converged Q-table, the computer mapped it to the reward map for the current maze. In the trial 2 to trial 8, the MFB stimuli were configured according to the reward map. If an unknown error branch in the maze were visited by the rat, the environment information of the maze would be updated immediately. The reward map would also be updated after the ratbot finished the trial.

The pipeline of the rule learning algorithm in T1 was shown in Fig. 6. Before the first trials of the task T1, there is no prior spatial knowledge about the maze for the ratbots or rats. After finishing the first trial, each of the six decision points has been traversed. Meanwhile, the computer got the navigation path by tracking the rat and a Q-table for the current maze was leaned by the Q-learning algorithm. The computer mapped the Q-value table to a digital reward map which is used to produce the stimulus intensity in the following seven trials. Consequently, there is no MFB stimulus for the rat in the first trial.

During the second to eighth trial, the digital reward map would be updated if the computer gets the spatial information for newly explored unit in the maze by tracking. The details are introduced in the following paragraphs in this subsection.

**Figure 7:**
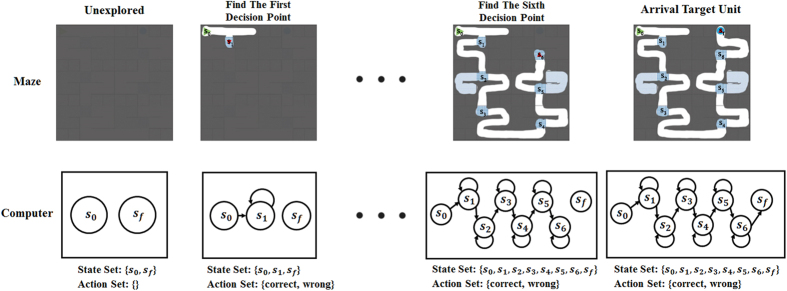
Maze environment information acquirement. The subgraphs in the top row represent the situations of maze exploration by the rat. The parts that are covered by gray denote the units which the rat has not visited yet, while the uncovered parts represent the units the rat has travelled. In the bottom row, the subgraphs show the environment information of the maze that the computer has got. As the number of the discovered decision points increasing in the top row, the relative state was added into the existing state set (i.e. the state *s*_1_ was added into the state set when the first decision point was found) initiated as {the start state *s*_0_, the final/target state *s*_*f*_}. And when the rat passed the first decision point, the action set was configured as {correct, wrong}.

Specifically, the computer tracked the rat’s position (*s*) and recorded the rat’s movement (*a*) in the maze. As shown in Fig. 7, the state *s* is divided by the six decision making points during the navigation path, which are the elements in the state set 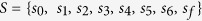 (*s*_0_: start state for the enter position, *s*_*f*_ : final state for the target unit, *s*_1_∼*s*_6_: the state for making the correct choice at ith decision point). The computer stored the traversed path and the obtained water rewards at the state *s*_*f*_. When the rats finishing the first trials, the state set *S*, the action set *A*, and the reward set *R* (the reward r for each state was set to 0 except the final state which is set to 100) were achieved. The problem was defined as a Markov decision process and solved with optimal policies by Q-learning algorithm (see Table 2).

**Figure 8:**
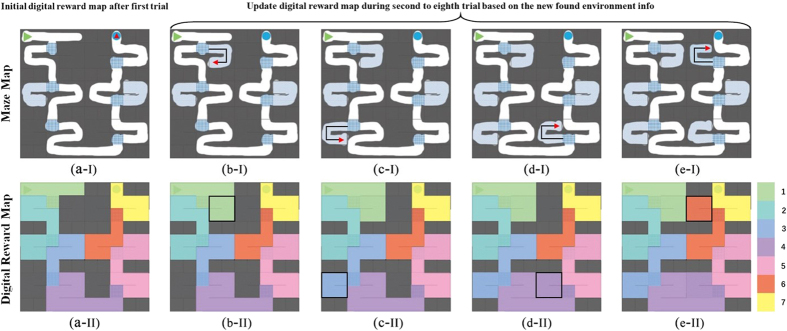
Digital reward map updating. During the second to eighth trial, once unknown units in the maze were visited by the rat, the digital reward map would be updated after the ratbot finished the trial. In (**b-I**), for the *i*th trial, the rat explored the unknown units’ trajectory denoted as the black line. The computer then updated the reward map to (**b-II**) before the (*i* + *1*)th trial. The updated area of the reward map was emphasized by the black box in the bottom row. Same situations were described in (**c–e**). The legend at the far right of the bottom row shows the mapping between colour and reward level (the number represent the reward level).

After got the converged Q-table, the computer mapped it to the digital reward map for the current maze and the rat by a log function. During the next few trials (2nd to 8th trial), the MFB stimuli were configured in accordance with this digital reward map. The detail of the method mapping the value in the reward map to MFB stimulus level was described in the following subsection “Graded level stimulation”. Once unknown units in the maze were visited by the rat, the digital reward map would be updated after the ratbot finished the trial. See Fig. 8b-I, for the *i*th trial, the rat explored the unknown units denoted as the black line. The computer then updated the reward map to Fig. 8b-II before the (*i* + *1*)th trial. Same situations were described in Fig. 8c–e.

When finishing all the sessions, the RULES algorithm was used to analyze the digital reward maps to extract the incremental gradient reward rule. The maze learning ability was enhanced by the levelled MFB stimulation intensities.

### Graded level stimulation

The smallest voltage of MFB stimulation that led to an observable behavioural response, and the maximum voltage that could be delivered without the rats showing signs of discomfort were tested. We referred to the two stimulation voltages as LowV and HighV respectively.

With the digital reward map computed from the reinforcement learning algorithm, the MFB stimulation intensities of different levels were calculated by the following three steps.

**Step 1:** Find the decision points in the digital reward map. The entry that had at least three adjacent units whose values were greater than zero would be a decision point. The number of MFB stimulation levels equaled the number of the decision points in the reward map plus 1.

**Step 2:** Sort the reward values at the decision points incrementally, denoted as 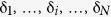.

**Step 3:** Translate the digital reward map into MFB stimulation levels. Given any entries δ′ in the map: If δ′ < δ_1_, the stimulation level was 1; If δ_*i*−1_ < δ′ < δ_*i*_, the stimulation level was n; If δ′ > δ_*N*_, the stimulation level was N + 1.

N + 1 stimulation intensities were distributed evenly in the interval between *LowV* and *HighV*.

## Additional Information

**How to cite this article**: Wu, Z. *et al.* Maze learning by a hybrid brain-computer system. *Sci. Rep.* **6**, 31746; doi: 10.1038/srep31746 (2016).

## Figures and Tables

**Table 1:** Experimental overview for the maze learning tasks.

Task	Subject Type	Subject Number	Session Number	Maze	Rules
T1	V1 Ratbot	6	16	no landmark	Incremental reward
T1 Control	Rat	6	16	no landmark	None
T2	V2 Ratbot	6	18	landmark	Landmark following
T2 Control	Rat	6	16	landmark	None
T2 with mask	V2 Ratbot with mask	6	18	landmark	Landmark following
T3	V2 Ratbot	5	15	landmark	Incremental reward and landmark following

**Table 2:** Q-learning algorithm for the maze learning.

**Input:**
Discount factor *γ*, Learning parameter α, Iteration times *t*
State Set (S): 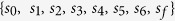
s_0_: start state, *s*_*f*_: final state, *s*_1_∼*s*_6_: the state of each decision point
Action Set (A): {correct, wrong}
Reward Set (R): the reward of each state was set to 0 except the final state which is set to 100
Exploration strategy: *ε-greedy*
**Process:**
initialize Q(*s*, *a*) to 0, 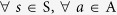
repeat
*s* is initialized as the starting state
set *t* to 0
repeat
choose an action 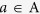 based on Q and the exploration strategy
perform action *a*
observe the new state s′ and received reward *r*
update Q value by: 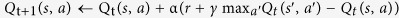
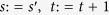
until *s*′ is a final state
until Q-value function was converged
